# A pan-cancer analysis of *MYC-PVT1* reveals CNV-unmediated deregulation and poor prognosis in renal carcinoma

**DOI:** 10.18632/oncotarget.9487

**Published:** 2016-05-19

**Authors:** Ioana Posa, Silvia Carvalho, Joana Tavares, Ana Rita Grosso

**Affiliations:** ^1^ Instituto de Medicina Molecular, Faculdade de Medicina, Universidade de Lisboa, 1649-028 Lisboa, Portugal

**Keywords:** PVT1, MYC, pan-cancer, KIRC

## Abstract

The *PVT1* lncRNA has recently been involved in tumorigenesis by affecting the protein stability of the MYC proto-oncogene. Both *MYC* and *PVT1* reside in a well-known cancer-risk locus and enhanced levels of their products have been reported in different human cancers. Nonetheless, the extension and relevance of the MYC-*PVT1* deregulation in tumorigenesis has not yet been systematically addressed.

Here we performed a pan-cancer analysis of matched copy number, transcriptomic, methylation, proteomic and clinicopathological profiles for almost 7000 patients from 17 different cancers represented in the TCGA cohorts. Among all cancers types, kidney renal clear cell carcinoma (KIRC) showed the strongest upregulation of *PVT1* and increased levels of both MYC and *PVT1* correlated with the clinical outcome. *PVT1* misregulation in KIRC is mostly associated to promoter hypomethylation rather than locus amplification. Furthermore, we found an association between MYC levels and *PVT1* expression, which impacted on MYC-target genes.

Collectively, our study discloses the role of *PVT1* as a novel prognostic factor and as a molecular target for novel therapeutic interventions in renal carcinoma.

## INTRODUCTION

Long non-coding RNAs (lncRNAs) play important regulatory roles in the gene expression and are deregulated in a variety of tumors [1]. The mechanisms through which lncRNAs contribute to the regulatory networks that lead to cancer development are diverse. lncRNAs can regulate gene transcription by binding promoter regions and/or changing histone marks and the chromatin state [2]. In addition, they may interact with and alter the activity of proteins, which may be important for cancer biology [3]. Recent findings revealed that the *PVT1* lncRNA controls MYC protein stability and they both cooperate to promote cell proliferation in cancer [4]. *PVT1* protects MYC protein from degradation by reducing the phosphorylation of a threonine residue [4].

*MYC* expression is complex and modulated at multiple levels but becomes deregulated in many human cancers. Interestingly, *MYC* and the *PVT1* lncRNA gene reside in 8q24, one of the most highly amplified locus across malignant tissues [5, 6]. Overall, MYC overexpression and *PVT1* up-regulation have been reported for several human cancers [7, 8]. However, the extension and relevance of *MYC* and *PVT1* alterations in tumorigenesis has not yet been thoroughly addressed.

In this study, we have integrated multi-omics data from The Cancer Genome Atlas (TCGA) to explore the relevance of *MYC-PVT1* deregulation across several cancer types. Our pan-cancer analysis revealed that kidney renal clear cell carcinoma (KIRC) shows the most extreme up-regulation of *PVT1* and the strongest connection between MYC*-PVT1* enrichment and clinical outcome. In KIRC patients, increased *PVT1* expression associated significantly with high MYC protein levels and misregulation of MYC responsiveness genes. Moreover, we found that *PVT1* up-regulation in KIRC is the result of promoter hypomethylation rather than copy number amplification. Altogether, our data disclose the prognostic power of the *PVT1* in KIRC and support its role as potential therapeutic target.

## RESULTS

### *PVT1* up-regulation in KIRC leads to poor survival

**Figure 1:**
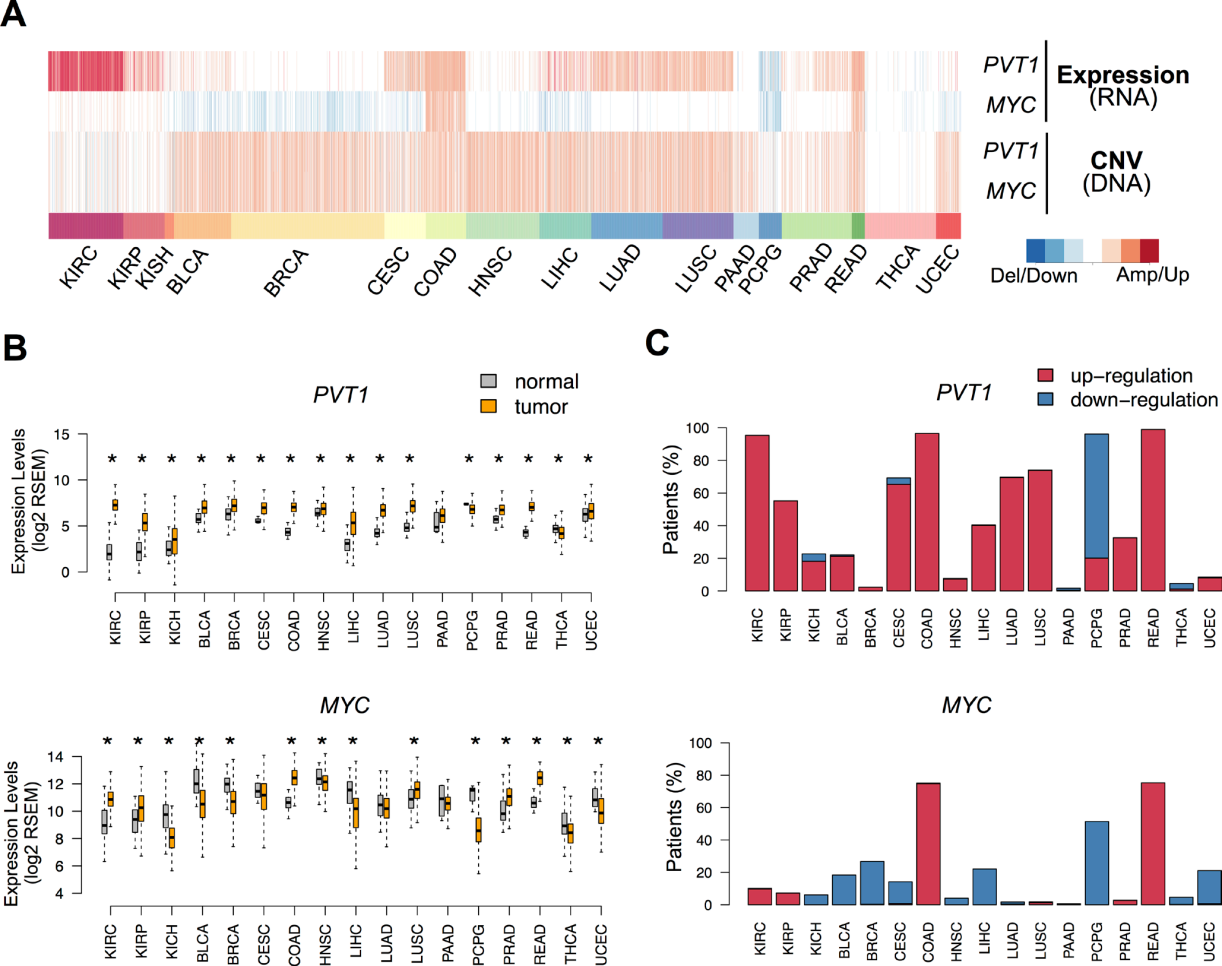
PVT1 and MYC deregulation in cancer (**A**) Heatmap with copy number variation and expression alterations for *MYC* and *PVT1* across 17 different TCGA cancer types: KIRC (kidney renal clear cell carcinoma), KIRP (kidney renal papillary cell carcinoma), KICH (kidney Chromophobe), BLCA (bladder urothelial carcinoma), BRCA (breast invasive carcinoma), CESC (cervical squamous cell carcinoma and endocervical adenocarcinoma), COAD (colon adenocarcinoma), HNSC (head and neck squamous cell carcinoma), LIHC (liver hepatocellular carcinoma), LUAD (lung adenocarcinoma), LUSC (lung squamous cell carcinoma), PAAD (pancreatic adenocarcinoma), PCPG (pheochromocytoma and Paraganglioma), PRAD (prostate adenocarcinoma), READ (rectum adenocarcinoma), THCA (thyroid carcinoma), UCEC (uterine Corpus Endometrial Carcinoma). Alterations are represented for each patient relative to normal tissue for expression (blue – down-regulation; red – up-regulation) and copy number variation (deletion – down-regulation; amplification – up-regulation). (**B**) *PVT1* and *MYC* expression levels (log2 RSEM) in normal (gray) and tumor (orange) tissues from patients. Significant differences are highlighted with * (Student *T*-test *p*-value < 0.05). (**C**) Proportion of patients with up-regulation (red) and down-regulation (blue) of *PVT1* and *MYC*.

We set out to investigate the impact of *MYC-PVT1* deregulation in several cancers using multi-omics data for approximately 7000 patients from the TCGA (Table S1). Overall, evaluation of copy number and transcriptome patterns showed distinctive *MYC-PVT1* profiles across the 17 cancer types inspected (Figure 1A). *MYC-PVT1* locus amplification was widespread and present in over half of the patients for most tumor types (Figure S1A). Hence, low frequency (less than 20%) of locus gain was observed for renal cancers (KIRC and KIRP), thyroid carcinoma (THCA), pheochromocytoma and paraganglioma (PCPG). Although widespread across cancers, the extensive locus gain was not mirrored by an overall up-regulation of both genes (Figure 1A). Increased expression levels of *PVT1* were observed for almost all tumors when compared to the surrounding normal tissues, with KIRC showing the largest difference (Figure 1B and S1B). A thorough analysis revealed a high prevalence of *PVT1* up-regulation in many tumors, spreading to 80% of the KIRC patients (Figure 1C). On the contrary, *MYC* misregulation differed according to the malignancy type (Figure 1B and S1B) and only colorectal cancers (COAD and READ) showed a high frequency of *MYC* up-regulation (Figure 1C). Furthermore, *PVT1* overexpression was also observed for several KIRC cell lines (Figure S1C). Although *PVT1* locus harbors several miRNA genes, their expression levels were similar between tumor and normal tissues (Figure S1D).

**Figure 2:**
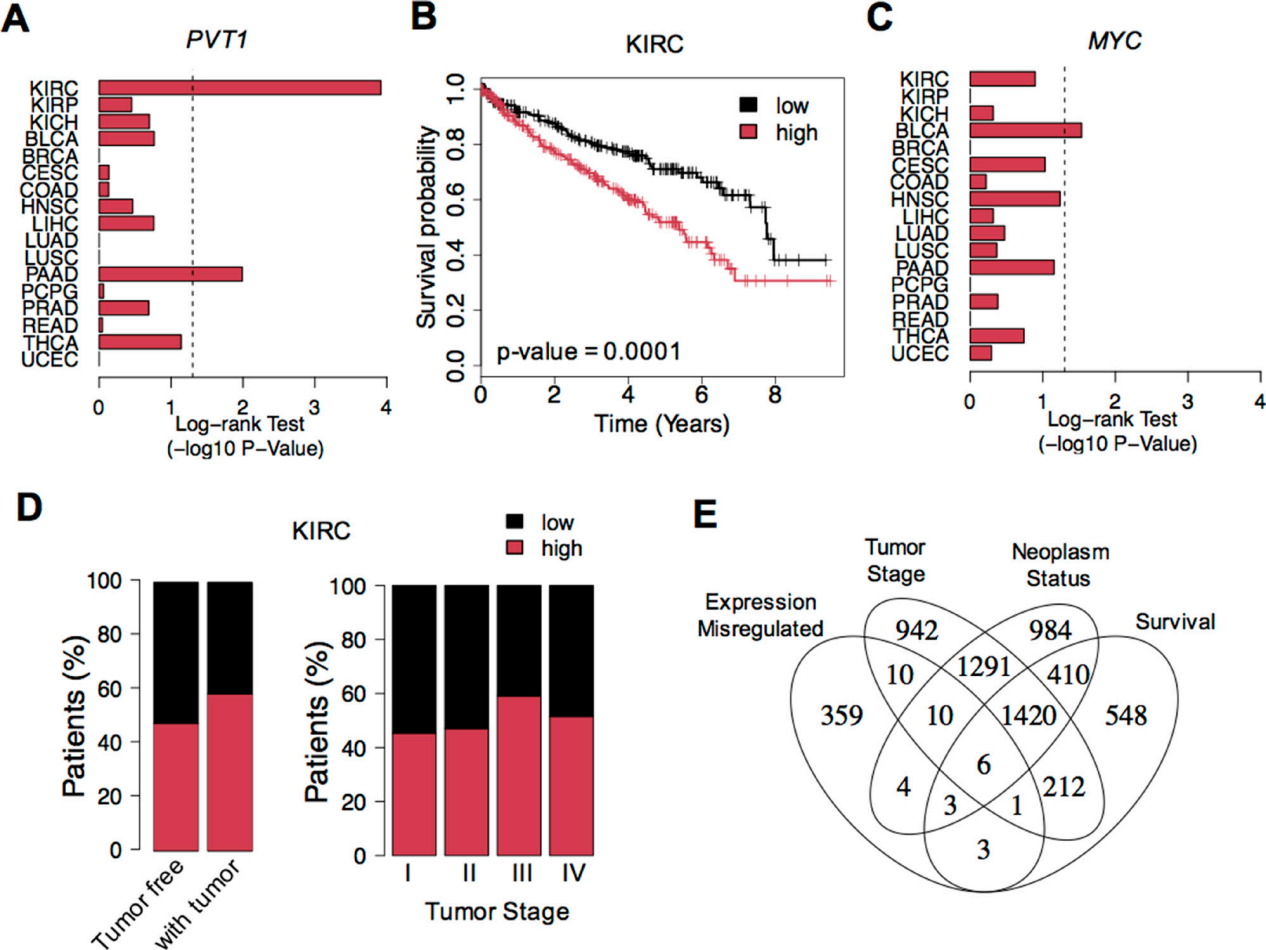
*PVT1* up-regulation and clinical outcome (**A**) Log-rank Test *p*-values (−log10 *p*-value) for survival analysis of *PVT1* expression levels across all cancers. The vertical dashed line corresponds to the significance level (*p*-value of 0.05). (**B**) Kaplan-Meier survival curves for *PVT1* expression in KIRC. Patients were split according to *PVT1* expression levels: high (red) and low (black). (**C**) Log-rank Test *p*-values (−log10 *p*-value) for survival analysis of *MYC* expression levels across all cancers. (**D**) Proportion of KIRC patients with high (red) and low (black) *PVT1* expression levels across different neoplasm status (tumor free and with tumor) and stages (I, II, III and IV). (**E**) Number of genes with significant expression alterations, association with neoplasm status, tumor stage and clinical outcome in KIRC. Six genes showed misregulation consistently associated with clinical features: PVT1, MYBL2, IL20RB for high expression; MFSD4, CRHBP and CWH43 for low expression in tumor samples.

Considering the widespread *PVT1* misregulation, we next assessed whether high expression levels would decrease the clinical patient outcome. Kaplan-Meier survival analyses revealed that *PVT1* overexpression was associated with worse survival rates in KIRC and pancreas adenocarcinoma (PAAD) (Figure 2A, 2B and S2A). High levels of *MYC* lead to low survival in bladder cancer (BLCA) (Figure 2C and S2B). Furthermore, supporting the impact of *PVT1* in the clinical outcome of KIRC patients, we observed that high expression levels were significantly associated with neoplasm status after surgery and advanced clinical stage or metastasis (Fisher's Exact -test *p*-value < 0.05) (Figure 2D). Interestingly, besides *PVT1*, only 5 other genes showed a strong misregulation consistently linked with clinical outcome and tumor features in KIRC (phenotype permutation *p*-value < 0.001, Figure 2E and Table S2). Most of these genes have been previously associated with worst survival or more aggressive state of tumors: *MYBL2* [9]; *IL20RB* [10]; *MFSD4* [11]; *CRHBP* [12] and *CWH43* [13]. These results indicate *PVT1* as a novel prognostic biomarker in KIRC.

### Promoter hypomethylation associated with *PVT1* up-regulation in KIRC

**Figure 3:**
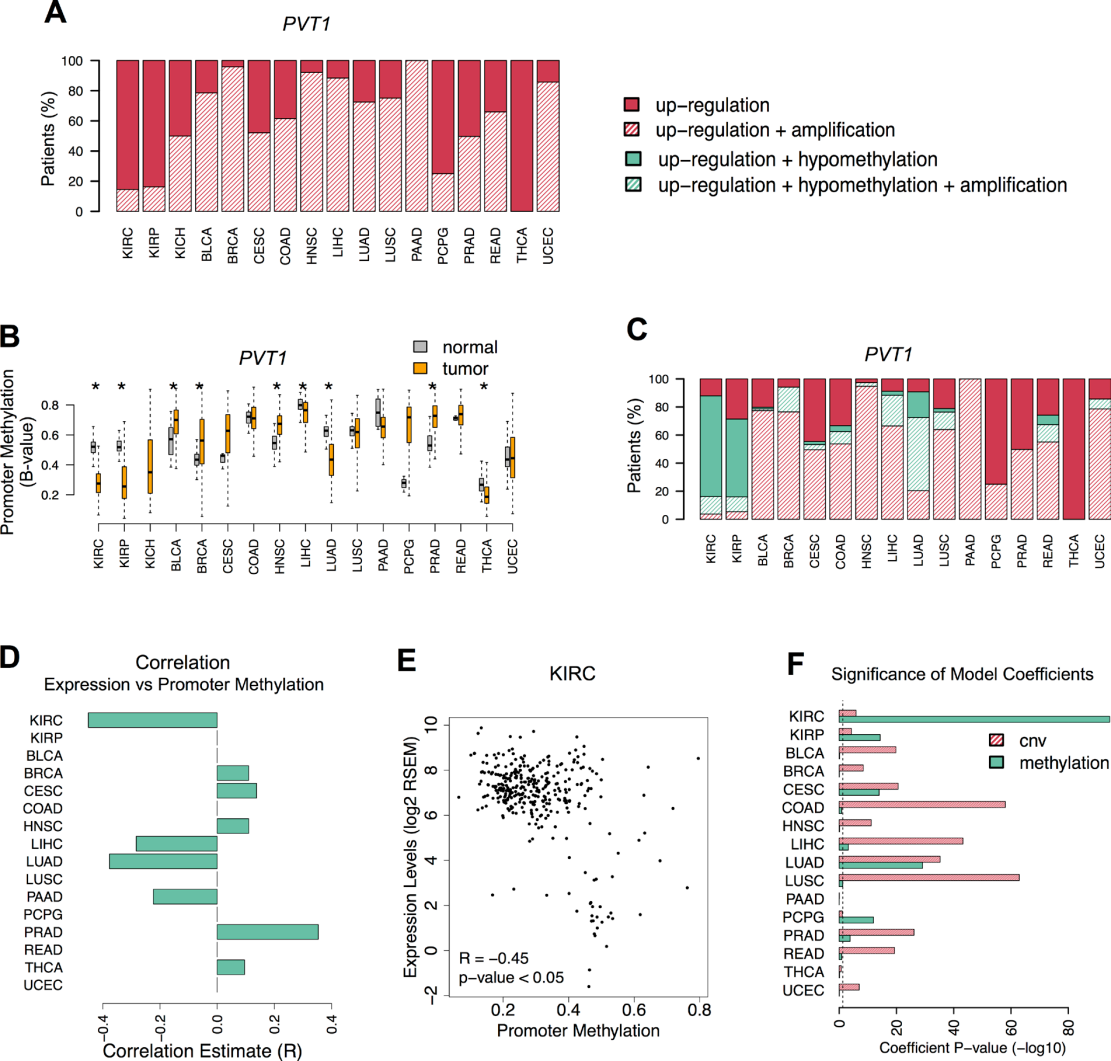
*PVT1* promoter hypomethylation (**A**) Proportion of patients with *PVT1* RNA up-regulation (red); RNA up-regulation and DNA copy number amplification (red stripes). (**B**) *PVT1* promoter methylation levels (B-value) in normal (gray) and tumor (orange) tissues from patients. Significant differences are highlighted with * (Student *T*-test *p*-value < 0.05). (**C**) Proportion of patients with increased levels of *PVT1* segregated in deregulated features: only RNA up-regulation (red); RNA up-regulation and DNA copy number amplification (red stripes); RNA up-regulation and DNA promoter hypomethylation (green); RNA up-regulation, DNA promoter hypomethylation and DNA copy number amplification (green stripes). (**D**) Pearson correlation estimates of significant association between *PVT1* expression and promoter methylation levels across all cancers (adj. *p*-value < 0.05). (**E**) Correlation between *PVT1* expression and promoter methylation levels in KIRC. (**F**) Significance of the model coefficients for *PVT1* copy number variation (red stripes) and promoter methylation (green) estimated for each cancer type. The vertical dashed line corresponds to the significance level (*p*-value of 0.05).

We next investigated which genomic alterations are the cause of *PVT1* increased levels across many different human cancers [5, 6]. A thorough analysis of individual profiles detected that *PVT1* locus amplification is observed in 40% of the patients from 13 cancers that have *PVT1* up-regulation. (Figure 3A). In contrast, *PVT1* up-regulation were not associated with locus amplification in 80% of KIRC patients. Besides copy number variations, misregulated gene expression in malignant tissues can be also driven by altered promoter methylation [14]. Thus, we subsequently assessed the methylation levels of *PVT1* promoter. Although all cancers showed alterations in *PVT1* promoter methylation, significant levels of promoter hypomethylation were found in KIRC (Figure 3B and S3A). Further analysis revealed that most KIRC patients with *PVT1* up-regulation also presented *PVT1* promoter hypomethylation (Fisher's Exact -test *p*-value < 0.005, Figure 3C). Indeed, *PVT1* expression levels were negatively correlated with promoter methylation in KIRC (R = −0.45, *p*-value < 0.05) (Figure 3D and 3E). Additionally, methylation profiles in KIRC cell lines showed overexpression of *PVT1* associated with promoter hypomethylation (Figure S3B). To estimate the effect of copy number alterations and methylation changes on *PVT1* expression levels, we fit a linear regression model to each TCGA cohort (Figure S3D). In general, *PVT1* locus amplification contributed significantly for *PVT1* misregulation in most cancer types (Figure 3F and S3E). However, in KIRC, promoter hypomethylation is a stronger determinant of *PVT1* up-regulation than locus amplification (Figure 3F and S3E). Overall, our results suggest that *PVT1* misregulation in KIRC is the result of promoter hypomethylation.

### Enhanced MYC protein and signaling is associated with poor prognosis in KIRC

**Figure 4:**
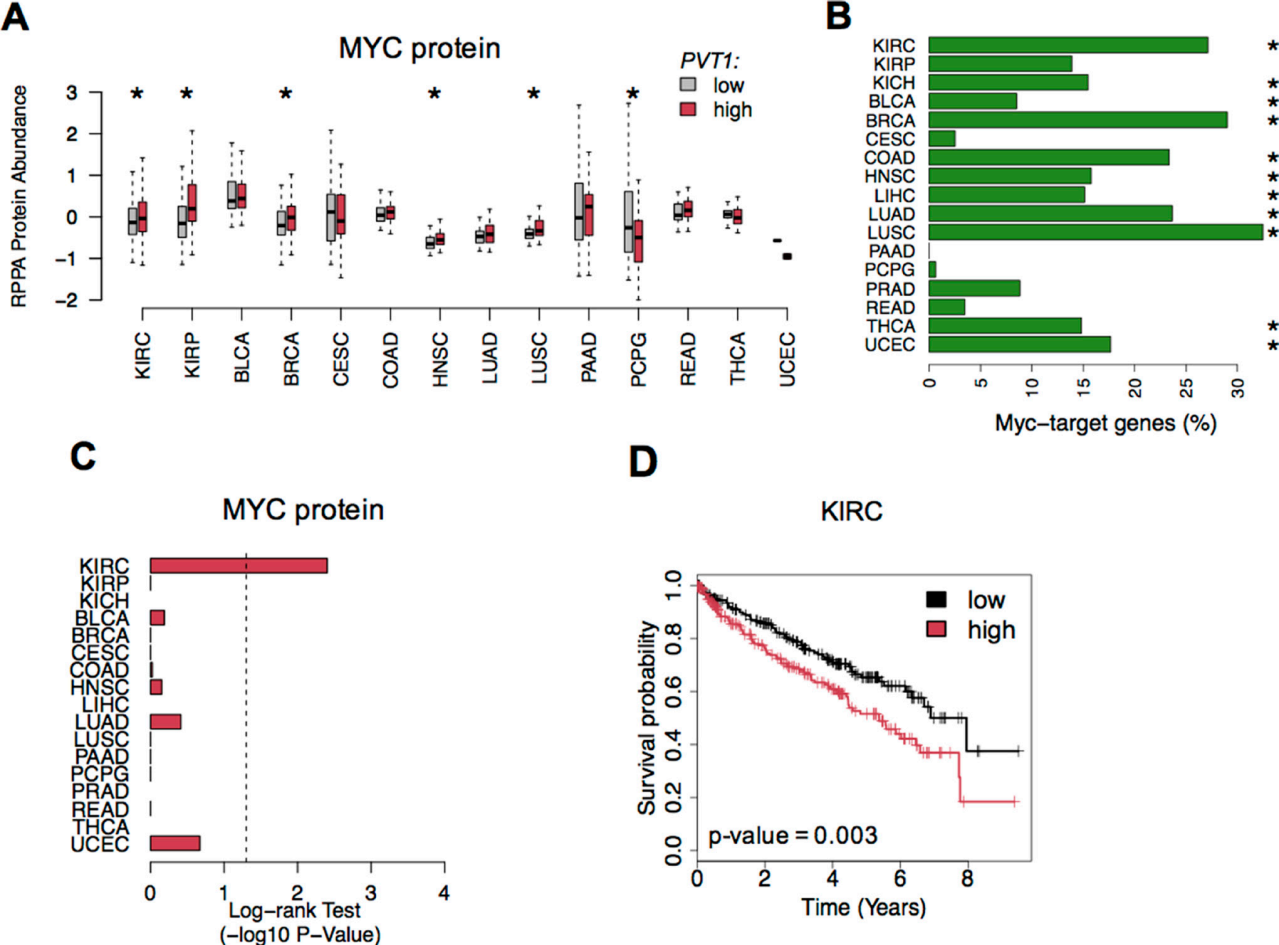
MYC protein levels in cancer (**A**) MYC protein levels (RPPA protein abundance) in tumor patients with low (gray) and high (red) *PVT1* expression levels. Significant differences are highlighted with * (Student *T*-test *p*-value < 0.05). (**B**) Proportion of MYC-target genes misregulated across all cancers. Significant enrichment is highlighted with * (Fisher's Exac*t*-test adj. *p*-value < 0.05). (**C**) Log-rank Test *p*-values (−log10 *p*-value) for survival analysis of MYC protein levels across all cancers. (**D**) Kaplan-Meier survival curves for MYC protein in KIRC.

Previous reports have shown that *PVT1* stabilizes MYC protein levels, promoting cell proliferation in cancer [4]. Thus, we assessed MYC protein levels using Reverse Phase Protein Array data (RPPA). We found that patients with high *PVT1* expression levels also showed a significant increase of MYC protein concentration for five cancers, including KIRC (Figure 4A). Because MYC is an oncogenic transcription-factor we then explored whether MYC-*PVT1* deregulation would impact genes responsive to MYC. Indeed, comparison of tumor and normal samples showed a significant proportion of MYC-target genes misregulated in KIRC and other cancers (Fisher's Exact -test adj. *p*-value < 0.05, Figure 4B and Table S3). Strikingly, only KIRC patients showed high levels of MYC protein associated with poor survival rates (Figure 4C and 4D). Further, 23% of the misregulated MYC-target genes in KIRC also showed a significant correlation with worse prognosis (Table S3).

Collectively, our results show that MYC-*PVT1* misregulation appears to be an important predictor of poor prognosis in renal carcinoma.

## DISCUSSION

Herein we performed a comprehensive analysis of almost 7000 patients from the TCGA to study the disrupted patterns of MYC-*PVT1* in cancer. Our pan-cancer analysis using diverse multi-omics data revealed that KIRC is the malignancy for which MYC*-PVT1* misregulation is most strongly associated with a poor overall survival.

Although *PVT1* gene is located in one of the most highly amplified locus across malignant tissues [5, 6], we found that less than 20% of the KIRC patients presented *PVT1* locus gain. Indeed, we further show that the increased *PVT1* lncRNA levels in KIRC patients are associated with *PVT1* promoter hypomethylation. Hence, our results suggest that promoter hypomethylation is an important cause of *PVT1* up-regulation in tumor patients lacking 8q24 locus amplification. Active DNA demethylation has been associated to the Ten-eleven translocation (TET) family proteins, which oxidize 5-methylcytosine into 5-hydroxymethylcytosine [15]. TET overexpression leads to promoter demethylation and transcription activation of specific genes [16, 17]. Since TET overexpression was already observed in different cancers [18, 19], such misregulation could be responsible for the hypomethylation of *PVT1* promoter in KIRC.

Recent findings revealed that gain of *PVT1* expression was required for high MYC protein levels in human cancer cells [4]. Indeed, our study reveals that KIRC patients show consistently high levels of both *PVT1* lncRNA and MYC protein, leading to misregulation of MYC responsiveness genes. Notably, increased levels of MYC protein and MYC-target genes are linked to worst prognosis in renal carcinoma. Thus, our data suggest that *PVT1* may act as an oncogene in renal carcinoma via stabilization of MYC protein, and subsequently activation of MYC pathway. However, future studies are required to confirm that high MYC protein levels in KIRC are caused by *PVT1*-induced MYC protein stabilization.

Inhibition of MYC is an attractive pharmacological approach for cancer treatment [20]. For instance, expression of a dominant-negative inhibitor of MYC heterodimerization in the mouse model for lung adenocarcinoma resulted in tumor regression [21]. However, MYC is an important transcription factor and an essential protein, thus therapeutic interventions to directly inhibit MYC have severe effects in patients [20]. Since loss of *PVT1* RNA in colon cancer cell line reduces MYC protein to more normal levels [4], inhibiting *PVT1* could be a more accessible and feasible therapeutic strategy for renal cancer. Modulation of lncRNAs functions have showed promising anticancer effects and expanded the development of lncRNA-based cancer therapies involving small interfering RNAs, antisense oligonucleotides, ribozymes and aptamers [22].

In conclusion, our study reveals that *PVT1* is strongly overexpressed in KIRC and associated to the enhancement of MYC signaling and worst clinical outcome. Moreover, our findings highlight the role of *PVT1* as biomarker for KIRC and a promising therapeutic target for cancer treatment.

## MATERIALS AND METHODS

### Large-scale data selection

The analysed TCGA data was downloaded from Broad Institute TCGA Genome Data Analysis Center (Table S1). Only cancers with transcriptome data for tumor and normal tissues (50 samples minimum) were selected, encompassing approximately 7000 patients from 17 different cancer types: KIRC (kidney renal clear cell carcinoma), KIRP (kidney renal papillary cell carcinoma), KICH (kidney Chromophobe), BLCA (bladder urothelial carcinoma), BRCA (breast invasive carcinoma), CESC (cervical squamous cell carcinoma and endocervical adenocarcinoma), COAD (colon adenocarcinoma), HNSC (head and neck squamous cell carcinoma), LIHC (liver hepatocellular carcinoma), LUAD (lung adenocarcinoma), LUSC (lung squamous cell carcinoma), PAAD (pancreatic adenocarcinoma), PCPG (pheochromocytoma and Paraganglioma), PRAD (prostate adenocarcinoma), READ (rectum adenocarcinoma), THCA (thyroid carcinoma), UCEC (uterine Corpus Endometrial Carcinoma). Number of normal and tumor tissues assessed by copy number variation (SNP Array); DNA methylation (Methylation BeadChip Array); expression (RNA-seq) and protein level (RPPA) are described in Table S1.

miRNA data for 568 KIRC patients (497 tumor and 71 normal) was obtained from TCGA in bam format. RNA-seq and BS-seq data for HEK293 [23] and KIRC cell lines [24, 25] were obtained from the GEO (http://www.ncbi.nlm.nih.gov/geo/, GSE68938, GSE51867, GSE64451, GSE44866).

### Copy number variation data analysis

Copy number levels from GISTIC algorithm represented locus loss (−2 for possible homozygous or −1 for heterozygous loss), locus gain (2 for possibly homozygous or 1 for heterozygous gain) and no alteration (0). Putative magnitudes of variations were considered for linear models analysis, whereas to determine patients with locus gain or deletion values were resumed to −1 (loss) or 1 (gain).

### Gene expression data analysis

TCGA expression data quantified as RSEM (RNA-Seq by expectation-maximization) [26] was logarithmically transformed (base 2) in order to follow a normal distribution. The statistical significance of differences in expression levels between normal and tumor samples was assessed using *limma* R Package [27]. To classify each patient based on gene expression misregulation, the expression of each tumor sample was compared to the distribution of normal samples for the same cancer type. Thus, a Z-score was calculated for each tumor sample and the statistical significance was assessed assuming a normal distribution. Finally, *p*-values were adjusted using FDR method to correct for multiple testing.

Expression levels from GEO RNA-seq datasets were obtained using Kallisto [28] and reference human genome (hg19).

TCGA miRNA expression levels were summarized (RPKMs) according to miRBase annotations [29] and differences were assessed using Student's *T*-test.

The statistical significance of survival differences in the Kaplan-Meier analysis was assessed using the Log-rank test and splitting the tumor samples in two groups: low and high expression levels (median value used as cut-off), as implemented in *survival* R package [30]. Different cut-off values were tested (Figure S4 and S5). Sample size calculation was performed using the Cox Proportional-Hazards Model implemented in powerSurvEpi R package (https://cran.r-project.org/web/packages/powerSurvEpi/) with postulated Hazard ration of 2 and statistical significance of 0.05. Despite the patient cohort heterogeneity, 11 (65%) cancers contained adequate sample size for the survival analysis (Table S4).

To identify genes with expression misregulation consistently associated with clinical features we used the following stringent criteria: 1) significant expression alterations in cancer (absolute log2 fold-change higher than 4 and FDR adj. *p*-value < 0.005); 2) survival (Log-rank Test FDR adj. *p*-value < 0.005); 3) neoplasm status (Fisher's Exact Test FDR adj. *p*-value < 0.005); 4) tumor stage (Fisher's Exact Test FDR adj. *p*-value < 0.005). For each association the direction of expression alteration and clinical outcome was considered, i.e. genes up-regulated in cancer should show high expression levels associated with worst survival, not tumor free and advanced clinical status (vice-versa for down-regulated genes). The significance of the expression-clinical associations was assessed using phenotype permutations, where the clinical features were randomly reshuffled and the procedures described above were recalculated on the reshuffled dataset. The process was repeated 1000 times recording the number of genes detected with expression-clinical associations.

Fisher's Exact-Test was used to evaluate association between the two groups and neoplasm status or tumor stage. MYC-target genes were obtained from Molecular Signatures Database [31, 32] and enrichment in differentially expressed genes was assessed using Fisher's Exact -test (*p*-values adjusted using FDR method).

### DNA methylation data analysis

TCGA methylation levels were obtained as Beta-values (using the intensity of the Methylated and Unmethylated Alleles), ranging between 0 (unmethylated) and 1 (fully methylated). Beta-values for microarray probes located in the promoter region (from 2 Kb upstream to 100 bp downstream of the TSS) were averaged to obtain the final promoter levels. Methylation levels from GEO BS-seq datasets were obtained using Bismark [33] and percent methylation was called averaging CpG sites from the promoter region.

The statistical significance of promoter methylation alterations between normal and tumor samples across cancers was assessed using Student's *T*-test. Promoter hyper/hypomethylation was determined using Z-score transformation and *p*-value correction, as done for expression data analysis. We used linear regression to model the *PVT1* expression in terms of its own copy number variation and promoter methylation. The performance of the model was estimated using analysis of variance. Correlation between gene expression and promoter methylation levels was assessed using Pearson correlation (*p*-values adjusted using FDR method).

### Protein data analysis

RPPA Protein abundance values were normalized using a z-score transformation. Tumor samples were split according to PVT1 expression levels (median value used as cut-off). The statistical significance of differences in MYC protein levels between patients with low and high PVT1 expression was assessed using Student's *T*-test. The statistical significance of differences in survival in the Kaplan-Meier analysis was assessed using the Log- rank test and splitting the tumor samples in two groups: low and high MYC protein levels (median value used as cut- off). Different cut-off values were tested (Figure S6). At least 79% of the cancers contained adequate sample size for the survival analysis (Table S4)

## SUPPLEMENTARY MATERIALS FIGURES AND TABLES




