# Supplementary material for: A pan-cancer analysis of MYC-PVT1 reveals CNV-unmediated deregulation and poor prognosis in renal carcinoma
Source: Oncotarget. 2016 May 19;7(30):47033–41. doi: 10.18632/oncotarget.9487 (PMC5216921; doi:10.18632/oncotarget.9487)
Supplement: Supplementary file 1 [file oncotarget-07-47033-s001.pdf]

A pan-cancer analysis of *MYC-PVT1* reveals CNV-unmediated deregulation and poor prognosis in renal carcinoma

Supplementary Materials

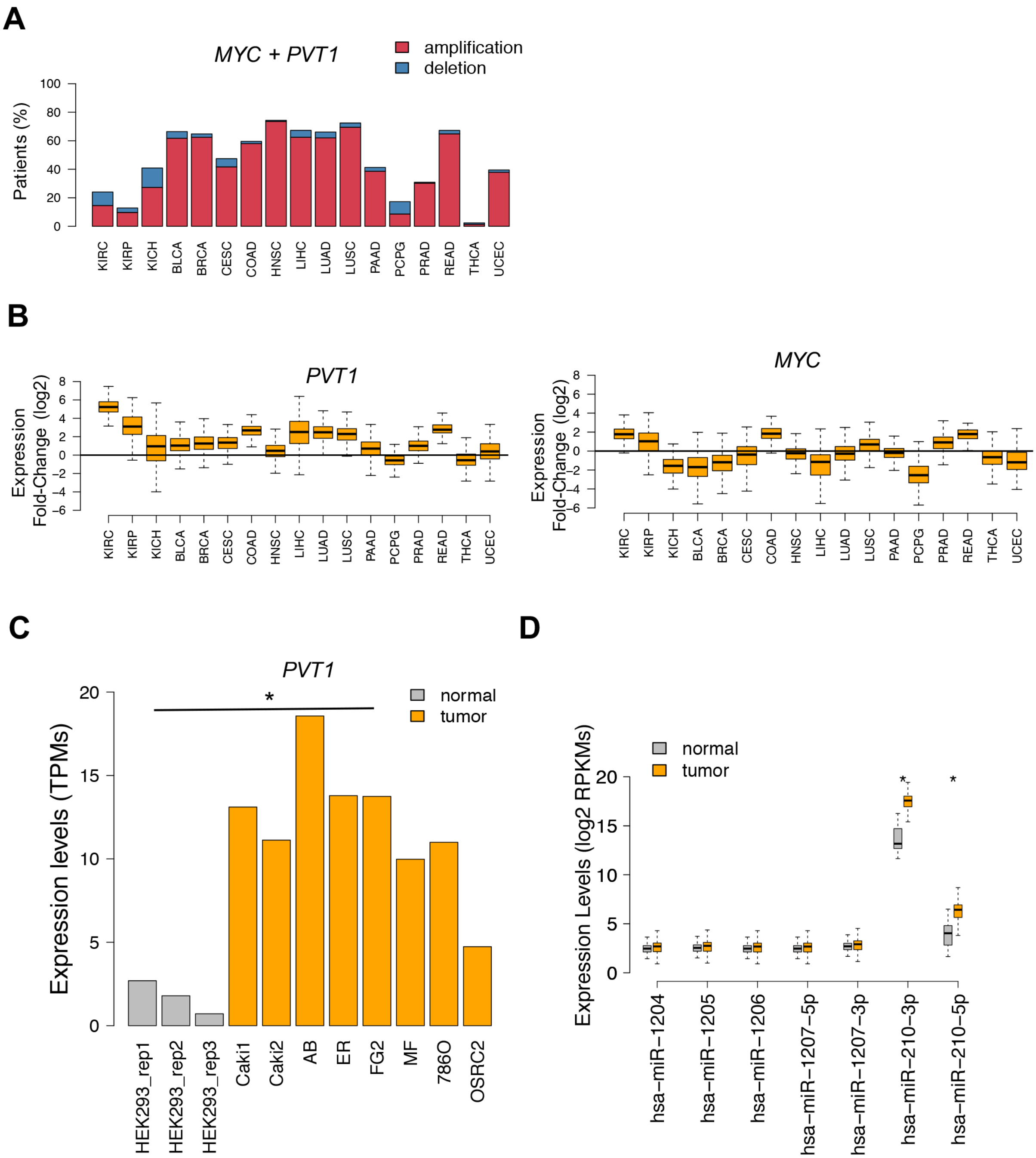

Supplementary Figure S1: *PVT1* and *MYC* deregulation in cancer. (**A**) Proportion of patients with copy number deletion (blue) and amplification (red) for *MYC-PVT1* locus. (**B**) *PVT1* and *MYC* expression alterations (log2 Fold-change) for each tumor sample relative to corresponding normal tissues. (**C**) *PVT1* expression levels in normal kidney and KIRC cell lines. (**D**) Expression levels (log2 RPKMs) of miRNAs genes transcribed from *PVT1* locus (has-miR-1204, has-miR-1205, has-miR-1206, has-miR-1207-5p, has-miR-1207-3p) and two miRNAs previously described with significant alterations in KIRC (Li M, Wang Y, Song Y, Bu R, Yin B, Fei X, Guo Q, Wu B. 2015. MicroRNAs in renal cell carcinoma: A systematic review of clinical implications (Review). *Oncol Rep* 33: 1571–1578). *Student's *T*-test *p*-value < 0.05.

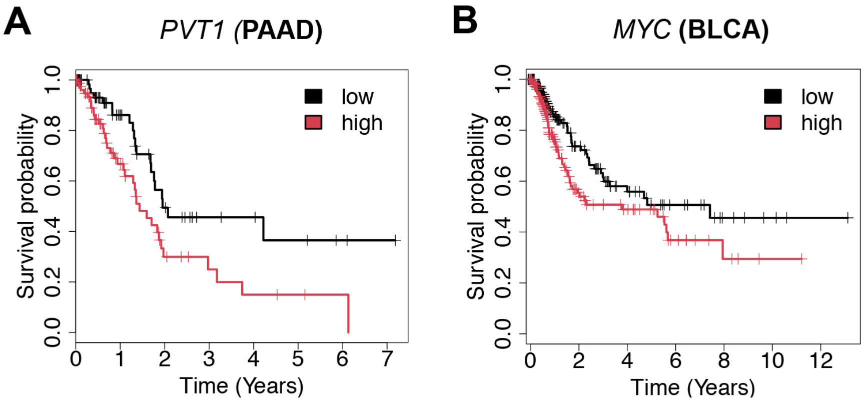

Supplementary Figure S2: Survival analysis. (**A**) Kaplan-Meier survival curves for *PVT1* expression in pancreatic adenocarcinoma (PAAD). Patients were split according to *PVT1* expression levels: high (red) and low (black). (**B**) Kaplan-Meier survival curves for *MYC* expression in bladder urothelial carcinoma (BLCA). Patients were split according to *MYC* expression levels: high (red) and low (black).

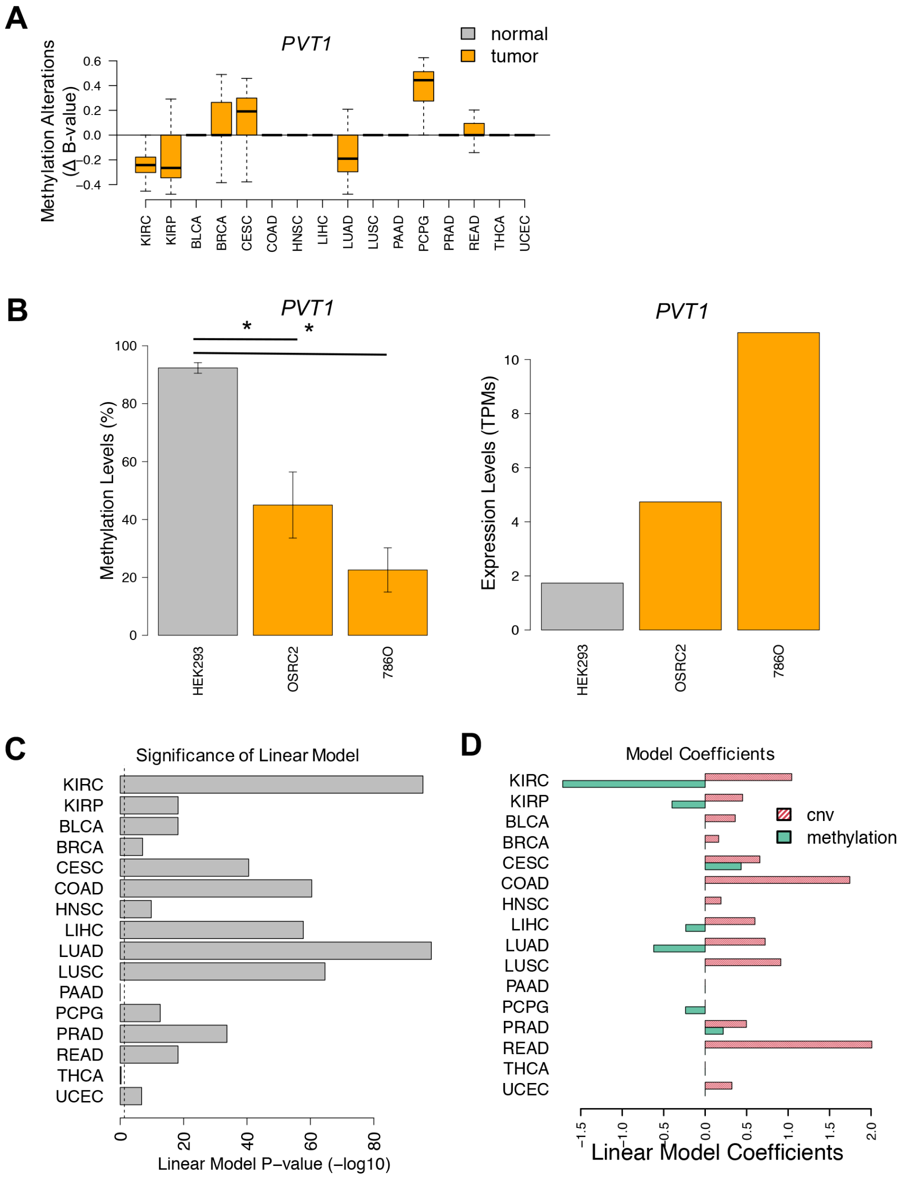

Supplementary Figure S3: *PVT1* promoter methylation. (**A**) *PVT1* promoter methylation alterations (Delta B-value) for each tumor sample relative to corresponding normal tissues. (**B**) *PVT1* promoter methylation and expression levels for normal and KIRC cell lines. Error bars represent standard error of the mean. *Student's *T*-test *p*-value < 0.05. (**C**) Significance of linear model the linear fitted for each cancer type. (**D**) Significant model coefficients for PVT1 copy number variation (red stripes) and promoter methylation (green) estimated for each cancer type.

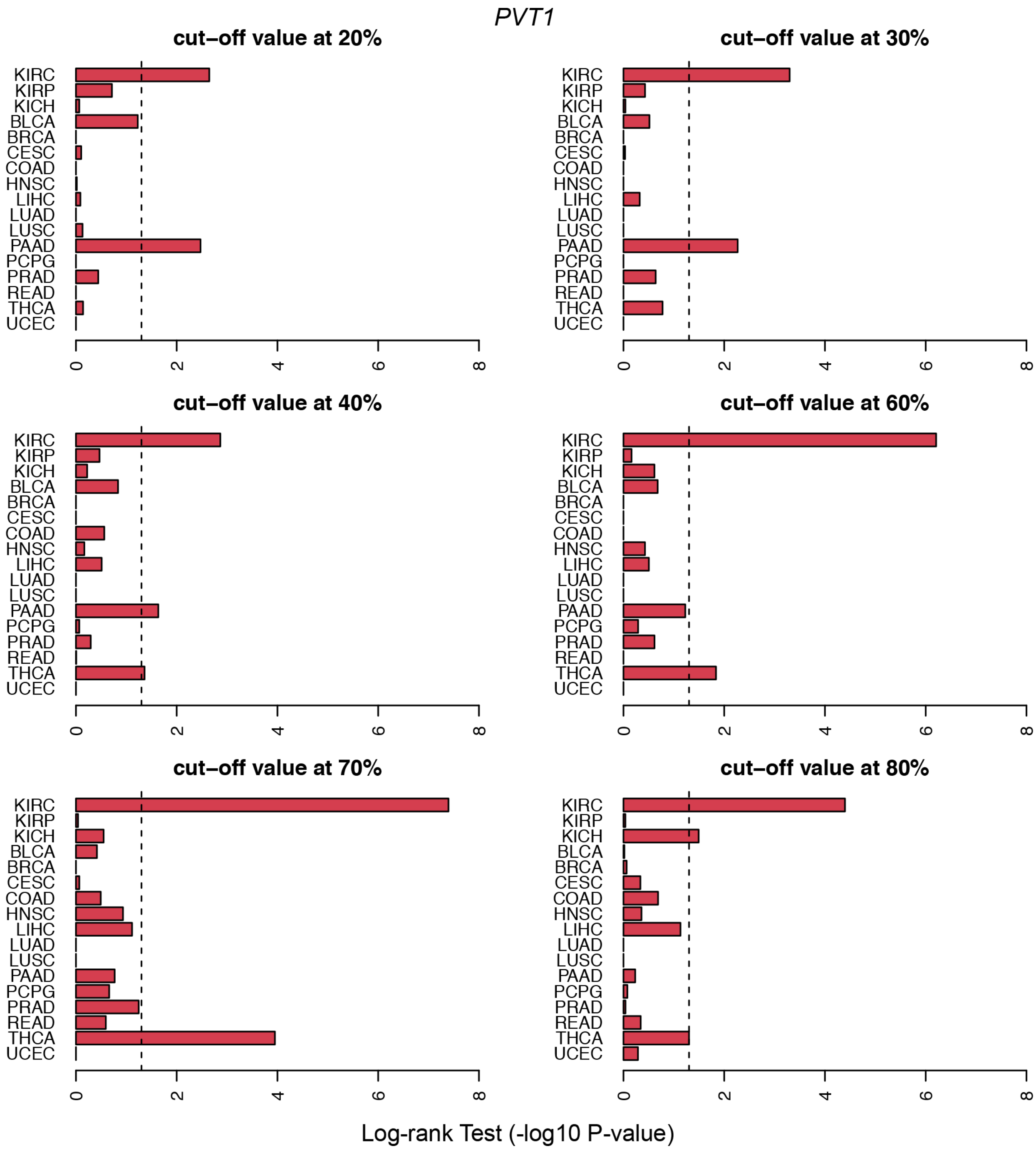

Supplementary Figure S4: Cut-off values of *PVT1* expression levels for survival analysis. Logrank Test *p*-values (-log10 *p*-value) for survival analysis of *PVT1* expression levels across all cancers. Patients were split according to *PVT1* expression levels using different cut-off values. The vertical dashed line corresponds to the significance level (*p*-value of 0.05).

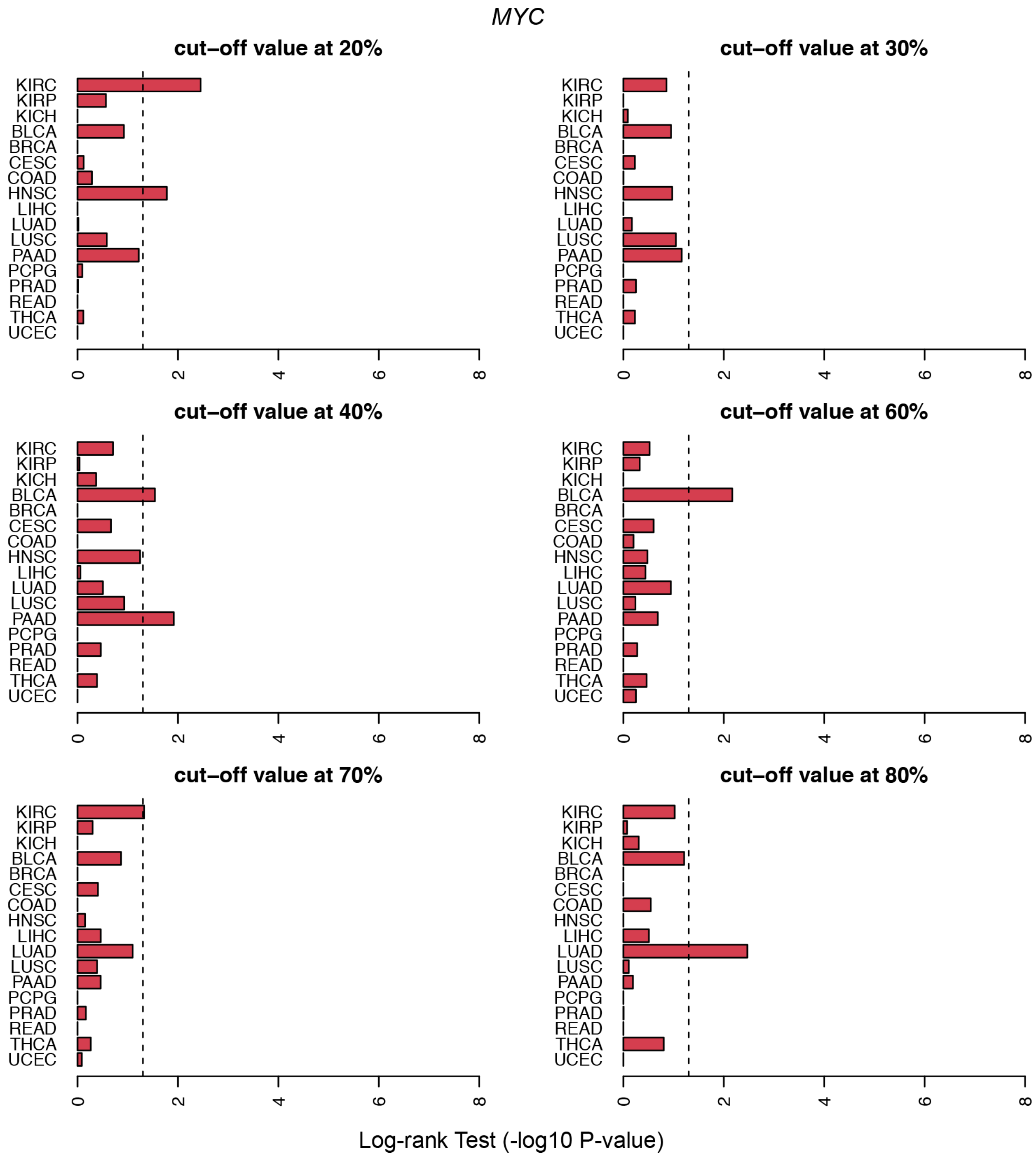

Supplementary Figure S5: Cut-off values of *MYC* expression levels for survival analysis. Logrank Test *p*-values (-log10 *p*-value) for survival analysis of *MYC* expression levels across all cancers. Patients were split according to *MYC* expression levels using different cut-off values. The vertical dashed line corresponds to the significance level (*p*-value of 0.05).

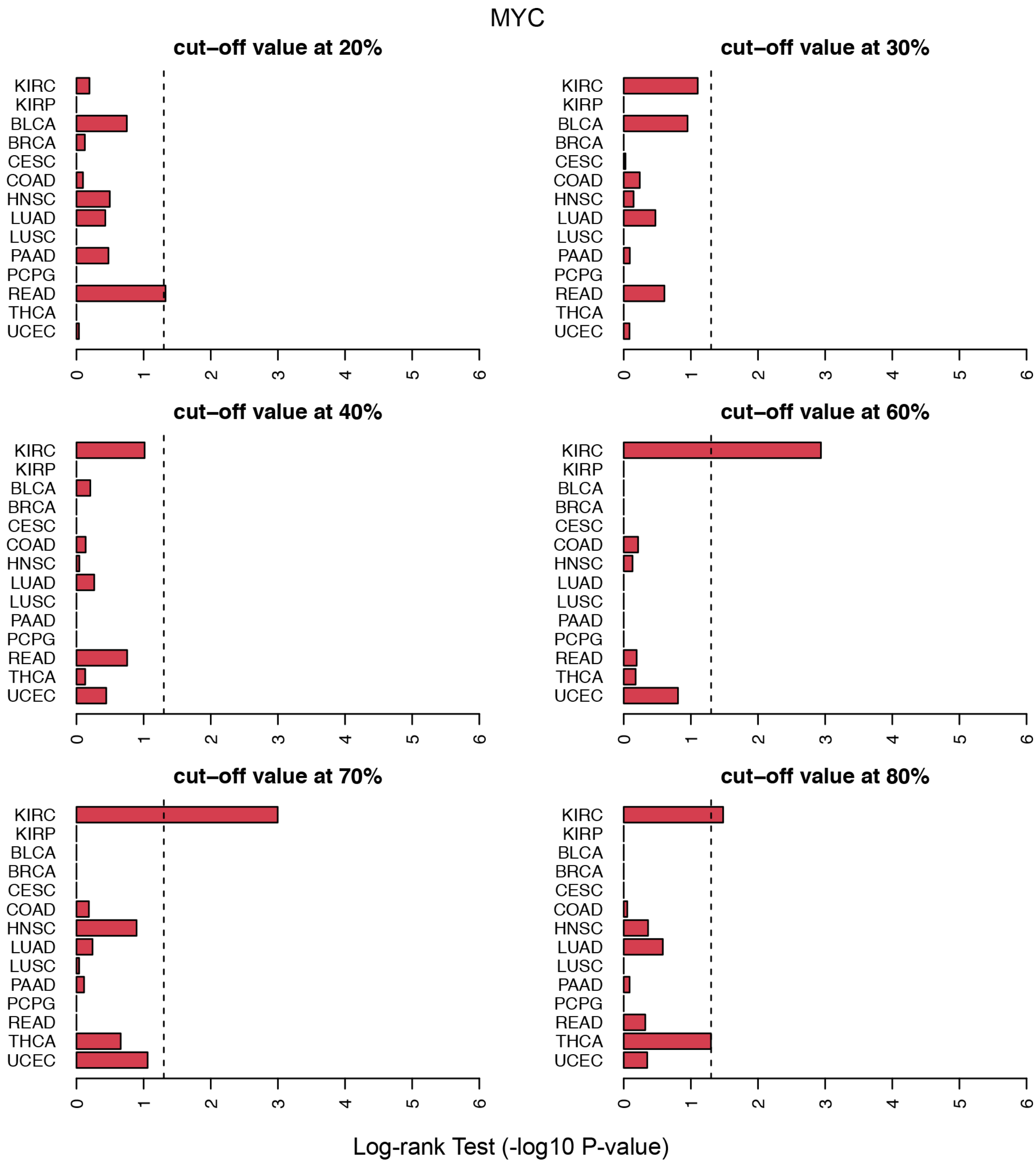

Supplementary Figure S6: Cut-off values of *MYC* protein levels for survival analysis. Log-rank Test *p*-values (-log10 *p*-value) for survival analysis of *MYC* protein levels across all cancers. Patients were split according to *MYC* protein levels using different cut-off values. The vertical dashed line corresponds to the significance level (*p*-value of 0.05).

Supplementary Table S1: The cancer genome atlas data

Cancers	Copy number variation	Methylation		Expression		Protein level (Tumor)	GISTIC Copy Number DOI
		Normal	Tumor	Normal	Tumor		
KIRC	528	160	320	72	534	454	doi:10.7908/C1RR1X8M
KIRP	288	45	276	32	291	208	doi:10.7908/C1TD9WD6
KICH	66	0	66	25	66	0	doi:10.7908/C13J3C06
BLCA	408	21	413	19	408	127	doi:10.7908/C1K0737C
BRCA	1080	97	772	112	1100	410	doi:10.7908/C1W37V8R
CESC	295	3	309	3	306	173	doi:10.7908/C1DR2TGB
COAD	450	38	297	41	287	331	doi:10.7908/C1K35SN9
HNSC	522	50	530	44	522	212	doi:10.7908/C1NK3D2T
LIHC	370	50	379	50	373	0	doi:10.7908/C1XK8DNT
LUAD	516	32	460	59	517	181	doi:10.7908/C1154G29
LUSC	501	42	370	51	501	195	doi:10.7908/C1JW8CZ4
PAAD	184	10	185	4	179	106	doi:10.7908/C18S4P1Q
PCPG	162	3	184	3	184	82	doi:10.7908/C13X85QT
PRAD	492	50	499	52	498	0	doi:10.7908/C1TB160T
READ	165	7	99	10	95	130	doi:10.7908/C1WW7GRD
THCA	501	56	511	59	509	224	doi:10.7908/C18W3CF1
UCEC	539	46	432	24	177	200	doi:10.7908/C1CC0ZTM

Number of clinical patients from The Cancer Genome Atlas assessed by copy number variation (SNP Array); methylation (Methylation BeadChip Array); expression (RNA-seq) and protein level (RPPA). Pre-processed data was obtained from Broad Institute TCGA Genome Data Analysis Center (2015): Firehose stddata__2015_04_02 run. Broad Institute of MIT and Harvard, doi:10.7908/C15X282W; SNP6 Copy number analysis (GISTIC2), GISTIC copy number DOI (see table).

Supplementary Table S2: Misregulated genes associated with clinical outcome and tumor features

Expression Misregulation			Survival		Tumor Stage		Neoplasm Status	
Gene Name	log2FC (TvsN)	Limma FDR adj. *P* value	Worst Prognosis	Log-rank Test FDR adj. *P* value	oddsRatio (High vs Low)	Fisher's Exact Test FDR adj. *P* value	oddsRatio (High vs Low)	Fisher's Exact Test FDR adj. *P* value
PVT1	5.15553545402218	1.10453254430704e-149	High Expression	0.00012045288814222	1.9899792	1.777711e-04	1.9920068	7.332912e-04
MYBL2	4.291371010761	5.70495102240996e-77	High Expression	4.68382695961722e-08	3.1887569	3.416804e-10	3.3404875	6.461962e-09
IL20RB	4.53540651820141	3.71417743841013e-33	High Expression	1.98195460043848e-10	3.8055094	6.338526e-13	3.0876141	4.192847e-08
MFSD4	–4.91265374909066	5.83542807043018e-151	Low Expression	1.17746529526563e-06	0.3984405	5.475386e-07	0.4555045	1.070530e-04
CRHBP	–4.58655950970351	7.98521758434403e-61	Low Expression	4.52943159645613e-08	0.3724151	7.850132e-08	0.4481283	7.363805e-05
CWH43	–5.74909164089697	1.69361342835474e-48	Low Expression	0.000144167607813217	0.4863079	8.557676e-05	0.5058515	7.498617e-04

Statistical ratios and significance for analyses assessing expression alterations, survival, tumor state and neoplasm status.

Supplementary Table S3: Misregulated genes across all cancer types Myc-target genes misregulated and Log Rank Test FDR adj. *p*-value for worst prognosis are indicated. See Supplementary_Table_S3

Supplementary Table S4: Sample size for survival analysis

Expression Data

Cancer	Original Sample Size	Sample Size Required		Adequate Sample Size
		PVT1	MYC	
KIRC	530	126	106	Yes
KIRP	271	171	186	Yes
KICH	66	286	218	
BLCA	389	134	146	Yes
BRCA	1083	209	292	Yes
CESC	303	128	142	Yes
COAD	280	216	138	Yes
HNSC	516	98	110	Yes
LIHC	359	108	108	Yes
LUAD	516	116	124	Yes
LUSC	492	102	112	Yes
PAAD	167	115	91	Yes
PCPG	184	468	300	
PRAD	488	2992	2006	
READ	95	402	202	
THCA	507	1284	910	
UCEC	167	243	326	

Protein Data

Cancer	Original Sample Size	Sample Size Required	Adequate Sample Size
		MYC	
KIRC	454	104	Yes
KIRP	205	183	Yes
BLCA	127	111	Yes
BRCA	410	172	Yes
CESC	172	130	Yes
COAD	331	89	Yes
HNSC	212	76	Yes
LUAD	181	99	Yes
LUSC	195	85	Yes
PAAD	104	84	Yes
PCPG	82	214	
READ	130	108	Yes
THCA	224	708	
UCEC	200	512	

Original and required sample size for survival analysis of PVT1 and MYC levels on each cancer type.
